# Phylogenetic diversity and cross-inoculation of indigenous isolated *Bradyrhizobium* from nodules of peanut in Liaoning province of China

**DOI:** 10.22099/mbrc.2019.32983.1392

**Published:** 2019-06

**Authors:** Hongzhi Bai, Yanhua Zhang, Haiqiu Yu, Muhammad Irfan, Yuqian Huang, Mei Han, Jinfeng Yang, Ning Liu, Hui Wang, Xiaori Han

**Affiliations:** 1 College of Land and Environment, Shenyang Agricultural University, Shenyang, China; 2 College of Agronomy, Shenyang Agricultural University, Shenyang, China; 3 Department of Biotechnology, University of Sargodha, Sargodha Pakistan; 4 College of Bioscience and Biotechnology, Shenyang Agricultural University, Shenyang, China

**Keywords:** Peanut, Bradyrhizobium, Phylogenetic diversity, Cross inoculation

## Abstract

*Arachis hypogaea.* L is a legume of economic importance, which is nodulated by *Bradyrhizobium, *a slow-growing bacteria. However there is no well characterization of this rhizobia in many areas of China. In the present study, cross-inoculation experiments were performed in cowpea and soybean. The isolated bacteria strains were characterized physiologyically, biochemically and identified through 16S rDNA sequence analysis showing that it belongs to *Bradyrhizobium japonicum*. The genetic diversity of the seventeen isolated strains were assessed through PCR-RFLP of 16S rDNA and 16S-23S rDNA IGS region. Cross inoculation test indicated that isolates could nodulate cowpea but not soybean. The cluster analysis based on physiological and biochemical characteristics showed the lower correlation between isolates and sites. The isolates were grouped into four clusters based on 16S rDNA gene sequence analysis. Thirteen polymorphisms were variable across all observations in 16S rDNA RFLP and six different IGS types from isolates. The results implies that there was some association between geographical factor and phylogenetic diversity of indigenous *Bradyrhizobium* isolates.

## INTRODUCTION

Rhizobia are nitrogen (N_2_)-fixing soil bacteria having symbiotic relationship with roots of leguminous plants. By this association, the total N_2_ contents in terrestrial ecosystem is of significant importance [1, 2] and is employed in sustainable agriculture, forestry and reforestry of degraded lands [3]. These symbiotic bacteria were divided into the *alpha-proteobacteria* (genera *Bradyrhizobium*), *Allorhizobium*, *Rhizobium*, *Mesorhizobium*, *Sinorhizobium* and *Azorhizobium* [4]. Recently, due to considerable taxonomic changes, rhizobia includes some members of the *beta-proteobacteria* such as *Cupriavidus *and *Burkholderia* [5]. *Arachis hypogaea* L. commonly known as peanut or groundnut, has great and important economical and agricultural potentials and is an importantand leguminous plant being used as food source in various food products. In this plant, nitrogen fixation is done through symbiotic rhizobia in root nodules. This plant is competent for intercropping with millet, sorghum, maize and other cereals due to its N_2_-fixation capacity, drought tolerance and shade tolerance [6]. China is a country with large scale export and import of peanuts in the world. The development of peanut industry has an important impact on the farmers' income, national food security and people's life improvment. Rhizobia inoculation improve the nitrogen fixation of peanut. However, the efficiency of symbiosis is influenced by the competition between the inoculated rhizobia and the native rhizobia [7], thus, selection of strains with the ability of nodulation, nitrogen-fixation and overcoming the competition witth the native strain is very important [8]. However peanut is widely cultivated in Liaoning, there is lack of information about the phylogenetic diversity of indigenous rhizobia. This investigation was aimed to characterize the native peanut rhizobia in Liaoning. Additionally, the population of rhizobia and the phylogenetic diversity of isolates were studied by analysis of the sequence divergence of the 16S rDNA, 16S-23S rDNA IGS region and Restriction Fragment Length Polymorphism (RFLP). Moreover, physiological and biochemical characterization of the isolates was carried out to assess the geographical variations which would be helpful for legume inoculation strategie [9,10]. The objective of this study was to find genetic diversity of rhizobia from different locations of Liaoning province of China in cross inoculation fields of soybean and peanut.

## MATERIALS AND METHODS

**Sampling of bacterial isolates: **The bacterial strains isolated from different source and geographic origins are described in Table 1. Samples were collected from field in the vicinity of root nodules of peanut plants from different locations in Liaoning province of China. For isolation of bacteria, the surface of nodules were sterilized in 95% ethanol for 30 s, followed by immersing in 0.1% mercury bichloride for 3-5 min. Then nodules were washed six times with sterile distilled water and plated on medium containing yeast mannitol agar [11] and incubated at 28 ℃ for 2-7 days. The pure culture was obtained after repeated streaking and stored at -80 ℃ using Yeast mannitol broth containing 20% glycerol. Growth rate and acid-base properties were observed on yeast extract mannitol agar (YMA) with bromothymol blue (BTB) plates to judge whether the isolates were *Bradyrhizobium *[12]. 

**Table 1:** The collection places of the isolates and the environment condition of the sites

**Cod** **e**	**Site**	**Location**	**Condition**	**Climate**
P,J	Fuxin	Western	Mountains and plains	Temperate semi arid and sub humid
B	Heishan	Western	Plains and hilly	Temperate continental monsoon
G	Xingcheng	Southwest	Hilly	Temperate monsoon
F	Kangping	North	Plains and hilly	Temperate continental
H	Faku	North	Plains and hilly	Temperate continental monsoon
O	Shenyang	Midland	Plains	Temperate sub humid continental
C,E	Liaozhong	South central	Plains	Temperate sub humid continental
4	Liaoyang	South central	Plains and hilly	Temperate sub humid monsoon

**Nodulation tests**: The isolated bacterial strains and the reference strain *Bradyrhizobium* strain1024 (from Institute of Applied Ecology: Chinese Academy of Sciences) were used for nodulation tests. The peanut seeds were surface sterilized according to Vanina et al. [11] and germinated at 28 ℃ using moist cotton in sterilized petri dishes, until the approximate length of radicle was 2.0 cm. Seedlings were sown in sterilized glass jars having an N-free nutrient solution (40% v/v) and vermiculite. Five seeds per jar and 1mL of bacterial suspension was used for the inoculation of the sown seeds and each experiment was conducted in five replicates. Controlled environment (light intensity of 200 µE·m^−2^s^−1^, 16 h day/8h night photoperiod, at 28 ℃ and 50% of relative humidity) was provided to grow the plants and regularly watered with applied N-free nutrient solution and sterilized tap water twice a month [11]. After 45 days of inoculation, plants were harvested and number of nodules were determined.

**Cross-inoculation tests: **Awareness of the benefits of cross-inoculation of native rhizobial strains with different host plants has increased in the past [4, 13]. Cross-inoculation experiments were performed in cowpea and soybean inoculated with the strains used in the study. The seeds were surface sterilized and sowed [11]. Harvesting of the plants were done 45 days after inoculation, and nodulation status were determined to evaluate if the strains had the ability of cross-nodulation.

**Physiological and biochemical characterization: **Physiological and biochemical characterization were performed by methods used for identifications of microbes [14, 15]. In this study, we characterized the isolates according to morphology, nitrogen fixing ability, hydrolyze amylum, IAA production, phosphate solubilization, intrinsic antibiotic resistance such as penicillin, erythrocin, streptomycin sulphate, gentamicin and kanamycin, salinity and acid-base properties resistance, dye resistance such as crystal violet and congo red, catalase and urease activity, keto-lactose and nitrate reduction reaction etc. The collected data were analyzed through NTSYS-pc for determination of polymorphism among bacterial strains.

**DNA Extraction: **DNA was extracted from fresh colonies of bacterial strains in exponential phase (0.4<OD600 nm<0.6), using bacterial genomic DNA extraction kit (TIANGEN, Beijing, China) following the protocol of the manufacturer.

**PCR-amplification and sequencing of the 16S rDNA:** After DNA extraction, Primers fD1(5′-CCG AAT TCG TCG ACA ACA GAG TTT GAT CCT GGC TCA G-3′) and rD1(5′-CCC GGG ATC CAA GCT TAA GGA GGT GAT CCA GCC-3′) were used for the amplification of the 16S rDNA gene. The PCR condition was adjusted as stated by Weisburg et al. [16]. The PCR products were run on 1% agarose gel electrophoresis. The amplified products were subjected to sequencing at Sangon Biotech, Shanghai, China.

**PCR amplification and RFLP of the 16S rDNA: **To screen the genetic diversity amongst isolates, PCR-RFLP analysis of 16S rDNA was employed. Primers fD1 and rD1 were used and protocol adopted for this is given in Table 2 with restriction enzymes *Hha* I, *Hinf *I*, Msp *I and *Hae* III, as reported by Laguerre et al. [17]. Restriction analysis was performed in total volume of 10 μL at 37℃ for 6 h, as specified by the manufacturer, and fragments were resolved on 3.0% agarose gel in 1× TAE buffer.

**Restriction fragment length polymorphism (RFLP) of the** **16S-23S rDNA IGS region: **The isolates were screened using restriction enzymes *Rsa* I, *Msp* I, *Hha* I and *Hae* III. Primers pHr（5′-TGC GGC TGG ATC ACC TCC TT-3′and p23SR01（5′-GGC TGC TTC TAA GCC AAC-3′）were used for the amplification of the 16S-23S rDNA IGS region. The protocol adopted is given in Table 2. The restriction analysis were performed in a total volume of 10μL for 6 h at 37℃.

**Table 2:** The PCR conditions used in this study

**Gene**	**Primer**	**Initial Denaturation**	**Denaturation**	**Anneling**	**Extension**	**Final Extension**
16S rDNA	fD1/rD1	94℃/3 min	94℃/30 sec	55℃/30 sec	72℃/1 min	72℃/5 min
	×30Cycles					
16S-23S IGS	pHr/p23SR01	93℃/3 min	94℃/1 min	57℃/45 sec	72℃/1.5 min	72℃/5 min
	×35Cycles					

**Phylogeny and** **Diversity analysis: **Homology analysis were performed via BLAST using NCBI search engine. MEGA (version 6.0) software was used for construction of phylogenetic tree [18] using neighbor-joining method [19]. The data collected from RFLP patterns were clustered based on unweighted pair group with mathematic average (UPGMA) with the Quantity One 4.52 and NTSYS-pc 2.11a.

This was compared with cross inoculation group which showed that rhizobia of peanut can nodulate cowpea but not soybean

## RESULTS

**Figure 1:**
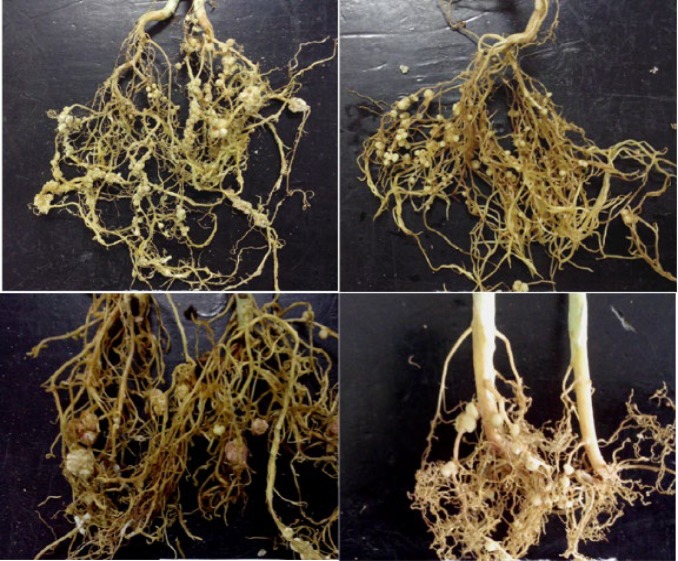
Nodulation status of parts strains on cowpea

After 45 days post-inoculation, the plants were harvested and nodulation status were determined. All isolates used in the study could nodule the peanut which proved that the isolates were rhizobia. Cowpea was nodulated effectively by the 17 rhizobia strains tested (Fig. 1). Soybean strains could nodulate none of the strains, non-inoculated plants did not have nodules. This was compared with cross inoculation group which showed that rhizobia of peanut can nodulate cowpea but not soybean.

**Figure 2:**
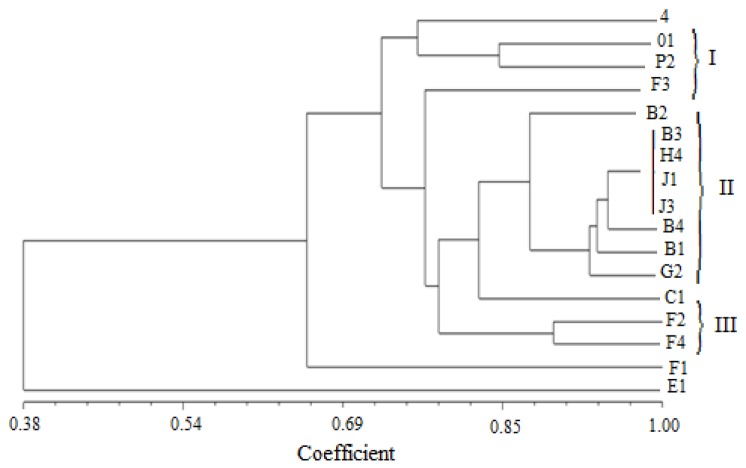
Dendrograms based on physiological and biochemical characteristics analysis using the UPGMA algorithm

All isolates were characterized based on morphological, biochemical and physiological characteristics. All the isolates had the same characteristics on some of targets, for example showing positive results onketo lactose and nitrate reduction reaction, while showed variable results between isolates in other tests, especially in intrinsic antibiotic resistance, salinity and acid-base properties resistance assays. For example, streptomycin sulfate resistance ranged from 0 μg·mL^-1^ to 100 μg·mL^-1^. We generated a dendrogram from the physiological and biochemical characteristics with NTSYS-pc analysis to identify the relative species. The dendrogram shown in Figure 2 revealed that the cluster analysis based on physiological and biochemical characteristics was divided into three groups with 80% level, while the isolates 4, F1, F3 and E1 did not cluster. Group II had most of the strains from different sites, all of strains from Heishan belonged to this group. Two isolates in group III were from the same site, but not all strains in this site. The cluster analysis based on physiological and biochemical characteristics showed the lower similarity between isolates and sites, but we could observe that majority of the similar strains belong to close sites**.**

**Figure 3:**
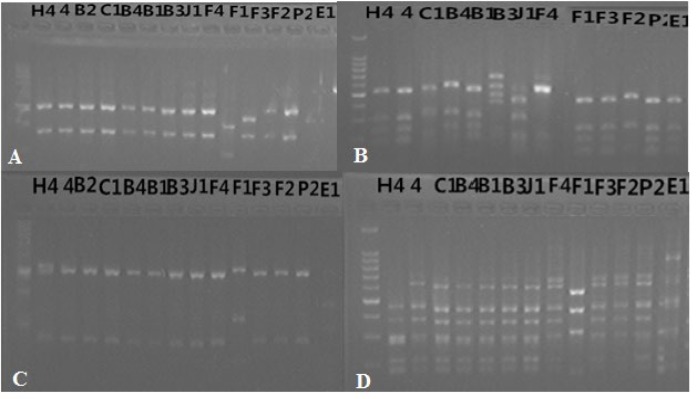
RFLP analysis of bacterial isolates by different restriction enzymes (A) *Hinf I *(B)* Msp I,* (C)* Hha I* (D) *Hae III*

PCR of the 16S rDNA gene from each isolate produced a fragment of approximately 1.45 kb. Using BLAST analysis, the 16S rDNA sequences of 12 typical isolates from different sites indicated that they belonged to the genus *Bradyrhizobium* with high similarity with *Bradyrhizobium japonicum* and deposited in GenBank under accession numbers from KR092316 to KR092327. RFLP analysis of 16S rDNA gene is a widely used tool for genetic studies of bacterial strains. In this research, *Alu *I*, Msp* I, *Hae *III, and *Hinf *I restriction endonucleases (Fig. 3) were used and thirteen polymorphisms were observed among isolates. Highest polymorphism (six different bands) was observed by *Msp *I endonuclease while *Hae *III, *Hinf *I produced five bands, and *Hha *I produced three different bands among tested bacterial strains (Table 3).

**Figure 4:**
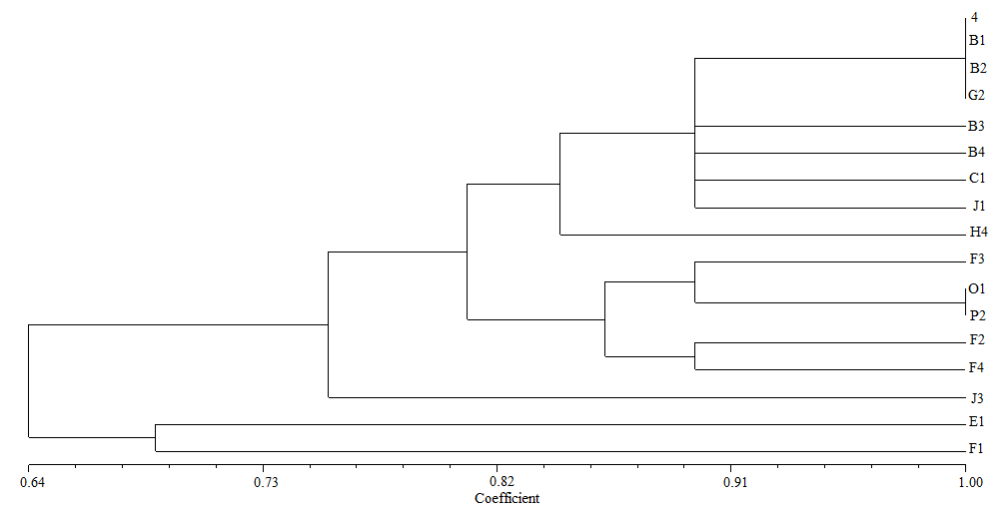
Dendrogram of bacterial isolates based on RFLP of 16S rRNA gene analysis

Genetic distance was studied by generating dendrogram from RFLP data among genotypes using the UPGMA algorithm. The dendrogram showed the distance of isolates from different sites (Fig. 4). According to sites, different restriction patterns were observed in isolates as compared to various sites, while similar RFLP band pattern indicate their association with specific sites, such as strains from Heishan. Moreover there were some strains in the same site belonging to different types, an exception was F1-F4 which were isolated from Kangping but it belonged to four different patterns and had lower similarity.

Amplification of the IGS region yielded a single band for all the 17 isolates. Digestion of the amplified IGS region of the isolates with the restriction enzymes *Msp *I*, Hae* III and *Rsa *I produced five restriction patterns, while *Hha* I revealed the lowest polymorphisms of IGS sequence. it produced three different band patterns. Six different IGS types were observed from the 17 isolates (Table 3).

**Table 3:** 16S rDNA and 16S-23S rDNA IGS PCR-RFLP patterns

**Denomination**	**16S rDNA PCR-RFLP**				types	**16S-23S rDNA IGS PCR-RFLP**				types
	*Msp*Ⅰ	*Hae *Ⅲ	*Hinf*Ⅰ	*Hha*Ⅰ		*Msp*Ⅰ	*Hae *Ⅲ	*Hha*Ⅰ	*Rsa*Ⅰ	
4	A	A	A	A	1	A	A	A	A	1
B1	A	A	A	A	1	A	A	A	A	1
B2	A	A	A	A	1	A	A	A	A	1
B3	D	A	A	A	2	A	A	A	A	1
B4	C	A	A	A	3	A	A	A	A	1
C1	B	A	A	A	4	B	A	A	B	2
E1	A	E	D	C	5	E	E	C	E	3
F1	A	D	B	B	6	C	B	B	C	4
F2	B	B	A	A	7	B	A	A	B	2
F3	A	B	C	A	8	D	C	B	D	5
F4	F	B	A	A	9	B	A	A	B	2
G2	A	A	A	A	1	A	A	A	A	1
H4	A	C	A	A	10	A	A	A	A	1
J1	E	A	A	A	11	A	A	A	A	1
J3	E	A	E	A	12	A	A	A	A	1
O1	A	B	A	A	13	B	A	A	B	2
P2	A	B	A	A	13	A	D	A	A	6

**Figure 5:**
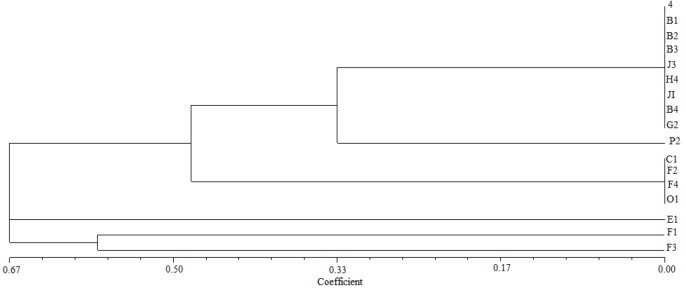
Dendrogram based on RFLP of 16S-23S IGS gene analysis using the UPGMA algorithm

The dendrogram obtained from combined RFLP and IGS patterns showed two main clusters (Fig. 5). Most isolates from one site had the same IGS pattern except strain P2. Most strains in the upper part of dendrogram belong to the western region, the rest of strains were isolated from central and northern areas, except the isolates from Kangping, which was related to three different patterns. The strain isolated from Fuxin was distributed into two IGS types. The result was similar with 16S rDNA RFLP, but there were some strains from different sites which had the same IGS pattern, that resulted in large diversity of isolates in Liaoning province. This illustrated that there were some association between geographical factor and phylogenetic diversity of indigenous rhizobium isolates.

## DISCUSSION

16S rDNA genes are often used as one of the markers of molecular systematics classification and evaluation phylogenetic relationship of rhizobia. In this study, 17 rhizobial strains were isolated from the peanut nodules from different peanut growing sites in Liaoning. The isolates in this study were showed the characteristics of genus *Bradyrhizobium* based on biochemical, physiological and molecular analysis and classified as genus *Bradyrhizobium*. In other words, *B. japonicum* was the dominant species of Liaoning province. 

 Generally, symbiotic nitrogen fixation of rhizobia and legumes was deemed to existin the phenomenon of cross inoculation group, but with the development of research on rhizobia, the concept began to be questioned. The study of Chang et al. [20] found that rhizobia of lentils and peanut can nodulate cowpea and peanut, similar to the results of peanut, rhizobia could nodulate cowpea in this study. Parveen et al [13] indicated that soybean rhizobia can nodulated cowpea, while Gillette and Elkan [21] have observed that *B. japonicum* can nodulate both soybean and cowpea. In this way, Li et al [22] indicated that parts of *Bradyrhizobium* of peanut can nodulate soybean, while some strains of soybean rhizobia can also make effective nodules in peanut, but the cross nodulation between these two kinds of rhizobia and legumes was not widespread. From previous reports, it was proven from cross-inoculation studies that rhizobia were shared by peanut and cowpea [13, 23,24]. This study provides evidence that peanut rhizobia can nodulate cowpea but cannot nodulate soybean. One explanation might be the physical and chemical properties of strains formed in the specific environment and symbiotic matching ability of soybean cultivars. 

The terrain of Liaoning was high in north and low in south, transition from land to the sea. It is possible to think that Liaoning province has approximately three geographical regions: the high lands in the west, plains in the middle, and hills in the east. So the divegence of terrain might affect the distribution of isolates. In this study, we generated a dendrogram based on physiological and biochemical characteristics to identify the relative species. Cluster analysis showed the lower similarity between isolates and sites. The isolates were identified in four clusters based on the 16S rDNA phylogenetic analysis. The genetic diversity among isolates was more considering that thirteen unique 16S rDNA RFLP types and six IGS types were obtained. 

Rhizobial strains could be analyzed using suitable restriction endonucleases particularly [25]. In this research *Alu *I*, Msp* I, *Hae *III, and *Hinf *I restriction endonucleases were used because of having good discrimination ability among peanut-nodulating strains [26,27]. Furthermore, each single type has association with a specific site except for F1-F4 which were isolated from Kangping but belong to different patterns and had lower similarity in both 16S rDNA RFLP types and IGS patterns very hard to understand what they wanted to say, while some isolates from different sites had the same IGS pattern. Previous data suggested that soil and climatic properties affect the diversity and distribution of indigenous rhizobia [11,28]. In Nepal the temperature and pH of soil significantly affect the distribution of rhizobia at species level in soybean [29]. Dinesh et al. [30] also reported the diversity of common bean nodulating rhizobia in different sites in Nepal. Chang et al. [20] used several methods, including 16S rDNA RFLP, IGS-RFLP, BOX-PCR and other’s for classified five strains of the genus *Bradyrhizobium* isolated from Sichuan and Anhui provinces of China. Ours results were in accordance with these reports.

Furthermore, the relative small sampling size from each site, could reduce the number of isolates and this diversity of *Bradyrhizobia* might have been taken too lightly. Most rhizobial isolates found in peanut nodules belong to the genus *Bradyrhizobium*. Although some other fast-growing effective rhizobia have also been reported [31-33]. A recent study described that RAPD, *glnII* , *recA* and nodC gene could illustrate the genetic diversity of bacterial strains isolated from root nodules of *Lupinus angustifolius* [34]. Salmi et al [35] reported that 16S rRNA, 16S-23S rRNA –ITS, *atpD*, *glnII* , *recA*  and nodC genes sequencing was very helpful for studying genetic diversity of *Bradizhobium* strains.
